# Preoperative CT-based radiomics combined with intraoperative frozen section is predictive of invasive adenocarcinoma in pulmonary nodules: a multicenter study

**DOI:** 10.1007/s00330-019-06597-8

**Published:** 2020-01-31

**Authors:** Guangyao Wu, Henry C. Woodruff, Sebastian Sanduleanu, Turkey Refaee, Arthur Jochems, Ralph Leijenaar, Hester Gietema, Jing Shen, Rui Wang, Jingtong Xiong, Jie Bian, Jianlin Wu, Philippe Lambin

**Affiliations:** 1 The D-Lab: Department of Precision Medicine, GROW - School for Oncology and Developmental Biology, Maastricht University, Maastricht, The Netherlands; 2 Department of Radiology, Affiliated Zhongshan Hospital of Dalian University, 6 Jiefang Street, Dalian, 116001 People’s Republic of China; 3 Department of Radiology, Maastricht University Medical Center+, Maastricht, The Netherlands; 4 Department of Radiology, The Fifth Hospital of Dalian, Dalian, People’s Republic of China; 5 Department of Radiology, The Second Affiliated Hospital of Dalian Medical University, Dalian, People’s Republic of China

**Keywords:** Carcinoma, non-small-cell lung, Machine learning, Frozen sections, Adenocarcinoma of lung, Tomography, spiral computed

## Abstract

**Objectives:**

Develop a CT-based radiomics model and combine it with frozen section (FS) and clinical data to distinguish invasive adenocarcinomas (IA) from preinvasive lesions/minimally invasive adenocarcinomas (PM).

**Methods:**

This multicenter study cohort of 623 lung adenocarcinomas was split into training (*n* = 331), testing (*n* = 143), and external validation dataset (*n* = 149). Random forest models were built using selected radiomics features, results from FS, lesion volume, clinical and semantic features, and combinations thereof. The area under the receiver operator characteristic curves (AUC) was used to evaluate model performances. The diagnosis accuracy, calibration, and decision curves of models were tested.

**Results:**

The radiomics-based model shows good predictive performance and diagnostic accuracy for distinguishing IA from PM, with AUCs of 0.89, 0.89, and 0.88, in the training, testing, and validation datasets, respectively, and with corresponding accuracies of 0.82, 0.79, and 0.85. Adding lesion volume and FS significantly increases the performance of the model with AUCs of 0.96, 0.97, and 0.96, and with accuracies of 0.91, 0.94, and 0.93 in the three datasets. There is no significant difference in AUC between the FS model enriched with radiomics and volume against an FS model enriched with volume alone, while the former has higher accuracy. The model combining all available information shows minor non-significant improvements in AUC and accuracy compared with an FS model enriched with radiomics and volume.

**Conclusions:**

Radiomics signatures are potential biomarkers for the risk of IA, especially in combination with FS, and could help guide surgical strategy for pulmonary nodules patients.

**Key Points:**

*• A CT-based radiomics model may be a valuable tool for preoperative prediction of invasive adenocarcinoma for patients with pulmonary nodules.*

*• Radiomics combined with frozen sections could help in guiding surgery strategy for patients with pulmonary nodules.*

**Electronic supplementary material:**

The online version of this article (10.1007/s00330-019-06597-8) contains supplementary material, which is available to authorized users.

## Introduction

Lung cancer ranks first in cancer mortality around the world [1]. With the popularization of computed tomography (CT) and the application of low-dose CT for lung cancer screening, substantial early-stage lung cancers have been detected [2]. Most malignant pulmonary nodules are confirmed as adenocarcinoma by pathology [3]. Patients with different types of adenocarcinoma differ in 5-year survival probabilities; e.g., patients with a diagnosis of invasive adenocarcinoma (IA) have a significantly poorer survival probability than those with adenocarcinoma in situ (AIS) or minimally invasive adenocarcinoma (MIA), who have a nearly 100% survival probability [4, 5]. Currently, lobectomy may be a better choice than sublobar resection for patients with IA, and patients with preinvasive lesions (atypical adenomatous hyperplasia (AAH) and AIS) and MIA (collectively PM) are candidates for limited resections [6].

Three methods are most commonly used to perform intraoperative or preoperative diagnosis in clinical practice, namely chest CT scan, biopsy, and intraoperative frozen section (FS). Many radiological studies rely on morphological (semantic) features such as spiculation or lobulation to generate a differential diagnosis. However, qualitative interpretation of the image is hampered by the strong subjectivity introduced by atypical radiology signs, especially in small and in ground-glass nodules [7–10]. Moreover, transbronchial and percutaneous biopsies are limited by the difficulties of sampling and localization [11]. FS has the potential to guide surgical strategy for peripheral small-sized pulmonary nodules by intra-operatively assessing adenocarcinoma type [6]. However, the coincidence rate of pathological diagnosis between frozen and paraffin specimens in early adenocarcinoma is hampered by sampling and interpretation errors, and by suboptimal specimen quality, due to the histologic heterogeneity of lung adenocarcinomas; e.g., it is difficult to identify whether the adenoid structure trapped around the scar is an invasive component or not [11–13]. The International Association for the Study of Lung Cancer emphasized that the diagnosis of adnocarcinoma cannot be firmly established without histologic sampling of the entire tumor. Review of CT images is recommended to add insights to the gross pathologic findings, which motivated the development of a CT-based radiomics model which combined with FS could further help distinguish IA from AIS or MIA [4].

Radiomics is the process that allows quantitative imaging features to be extracted in bulk, creating unique fingerprints for images (or regions of interest (ROI) therein) which can be correlated with clinical data using machine learning approaches [14, 15]. Recently, CT-based radiomics have shown excellent predictive performance to differentiate IA from PM of the lung [16–19]. Therefore, this study aimed to develop and validate a multicenter multifactorial radiomics model combined with FS results and clinical parameters to distinguish IA from PM in pulmonary nodules.

## Materials and methods

### Patients

The institutional review boards approved this retrospective study registered in http://clinicaltrials.gov (identifier: NCT03872362), and the requirement for informed consent was waived. A total of 582 patients with 623 nodules underwent lung operation and non-contrast-enhanced CT scans between January 2013 and October 2017. The patient cohorts from three centers were identified according to the established inclusion and exclusion criteria. The inclusion criteria were (1) primary lung adenocarcinoma, confirmed by pathology and (2) pulmonary nodules without a visible cavity, which would either alter feature values or otherwise complicate the delineation process. The exclusion criteria were (1) previous history of radiation therapy, chemotherapy, or biopsy before baseline CT scan; (2) the time interval between the CT examination and surgery was more than 2 weeks; and (3) insufficient CT or pathology quality to make a diagnosis. Clinical information was procured from medical records.

**Fig. 1:**
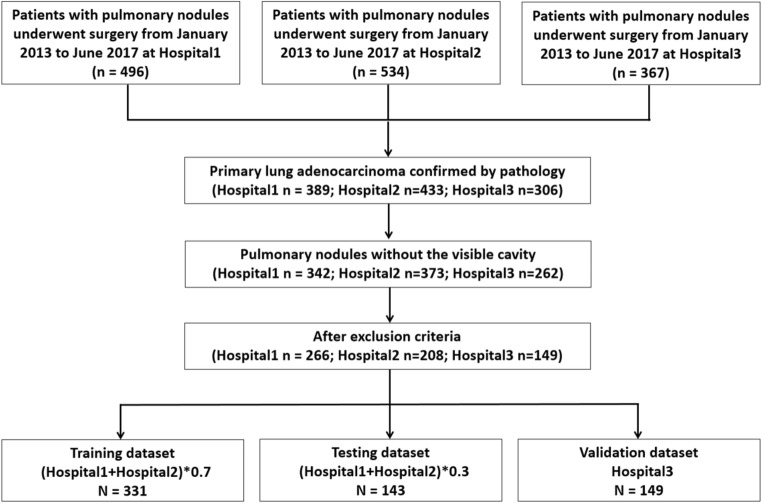
Flowchart for patient selection from three hospitals

All nodules from Hospital1 and Hospital2 were aggregated and randomly divided into two datasets, 70% for the training dataset and 30% for the testing dataset, while attempting to maintain the original class balance in the sub-cohorts. All nodules from Hospital3 were allocated to the external validation dataset (Fig. 1).

### Image acquisition, semantic features, and pathological evaluation

CT scans were performed on a 64- or 128-detector row CT system (Somatom Definition, Siemens Medical Solutions) with the following acquisition and reconstruction parameters: tube voltage 100 kV or 120 kV; the tube current is automatically calculated; pitch 0.75–1.5; collimation 0.6 mm; matrix 512 × 512. The reconstruction algorithm of “bone plus” for thin-section helical scans was used with a thickness of 1.0–1.5 mm. Further detailed acquisition parameters are provided in Supplementary S1.

Two chest radiologists each with 8 years of experience were blinded to evaluate the images in the lung window setting (window, − 600 HU; width, 1200 HU) and the mediastinal window setting (window, 40 HU; width, 350 HU). The type of ground-glass nodule (GGN) and solid nodule was defined as well as the lesion diameter was reported according to the guidelines from Fleischner Society [20, 21]. FS and final pathology results were a blind assessment by two pathologists according to the International classification of lung adenocarcinoma [4]. Atypical adenomatous hyperplasia (AAH), adenocarcinoma in situ (AIS), and MIA were categorized as PM. Cases of disagreement for semantic features and pathological assessments were resolved through consultation.

### Segmentation

**Fig. 2:**
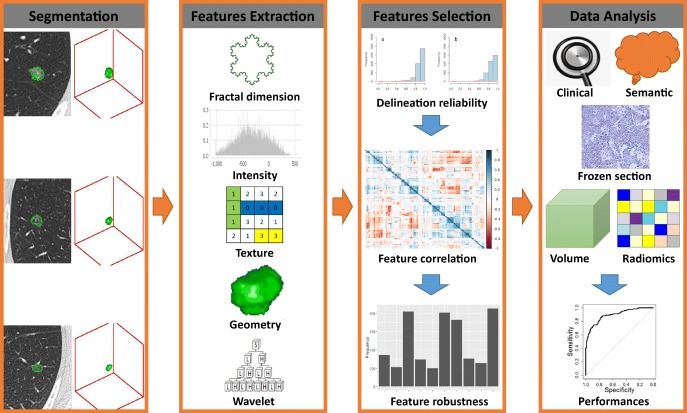
Flowchart showing the process of radiomics

The workflow of radiomics from segmentation to data analysis is shown in Fig. 2. The ROI was manually contoured slice-wise on the axial projection to arrive at a 3D-segmentation using MIM (MIM Software Inc.). One radiologist with 8 years of experience performed segmentation for all cases. To assess intra-reader agreement, 50 randomly selected cases were segmented again by the same radiologist, as well as by another medical doctor with 3 years of contouring experience. Apart from the volume feature computed by the radiomics software, tumor volume was also computed by MIM after contouring.

### Image processing and feature extraction

All images were resampled to an in-plane pixel spacing of 0.75 mm and a slice thickness of 1.5 mm using linear interpolation to partially counter the heterogeneous reconstruction settings found in the database [22]. For non-filtered features, excluding first-order statistics features, voxels values, represented in Hounsfield units (HU), were aggregated into bins of 25 HU wide in order to reduce noise and inter-scanner variability. Filtered features used a fixed number of bins equal to the number calculated for non-filtered features. Feature extraction was performed using the RadiomiX Discovery Toolbox (OncoRadiomics SA). The features extracted describe fractal dimension, intensity histogram, first-order statistics, texture, local intensity, shape, and features extracted from wavelet-filtered images. Descriptions and mathematical definitions of the features have been described in detail previously [23].

### Feature selection

The intra-/inter-class correlation coefficient (ICC) was used to assess the robustness of features between the individual radiologist contours. Features with ICC values < 0.8 were removed from further analysis. Features with little variance across the cohort (the ratio of the frequency of the most common value to the frequency of second most common is greater than 95/5) have little explanatory value and hence were removed. Likewise, highly correlated features needlessly inflate the dimensionality of feature space. For feature pairs with a high Spearman correlation (*r* > 0.8) in the data from Hospital1 and Hospital2, the feature with the highest mean correlation with all remaining features was removed. The cases from Hospital1 and Hospital2 were randomly spilt into training (70%) and testing (30%) datasets 1000 times. For each iteration, the top ten features were ranked and selected in the training dataset using recursive feature elimination with the *treebag* method and a cross-validation technique (tenfold, 10 times), and a random forest model built in the training dataset using the top 5 features and evaluated in the testing dataset. The features with the highest selection frequencies in the 1000 iterations were retained. Finally, features with high Spearman correlation with volume (*r* > 0.8) were removed since volume is examined as an independent feature in univariate and multivariate analyses.

### Model training and validation

Random forest binary classification models were trained using an increasing number of features from the previous step, starting with the highest ranked feature, and their performance was tested on the testing dataset until the area under the curve (AUC) of the receiver operator characteristic (ROC) increased < 0.02 in order to strike a balance between good performance and possible overfitting. The final model (with a set number of features) is trained on the combined training and testing dataset and validated on the external dataset. The radiomics model was developed using CT-based radiomics features, the clinical model was created based on clinical variables (age, gender, smoking status, and family history of lung cancer), and the semantic model was built with semantic features (location, diameter, and nodule type). Lesion volume values were used to build the volume model. The radiomics model combined with volume was named RV, which was further combined with clinical and semantic information (CSRV). FS results were combined with the radiomics and volume to build a multifactorial model (FSRV), and likewise, volume was added to the FS model (FSV). Finally, all data including clinical variables, semantic features, FS, radiomics features, and tumor volume were used to build a combined model called CSFSRV. In order to examine the entire pipeline for the ability to find spurious correlations, the outcomes were randomized and the process repeated, including feature selection and model building.

### TRIPOD guidelines, radiomics quality score, and statistical analysis

This study was followed by the Transparent Reporting of a Multivariable Prediction Model for Individual Prognosis or Diagnosis (TRIPOD) guidelines [24]. The radiomics quality score (RQS) was used to evaluate the radiomics workflow [25].

Pearson’s chi-squared test was used for the statistical analysis of essential demographic characteristics. The performances of the random forest binary classification models were evaluated with receiver operating characteristic (ROC) curve to calculate the area under the curve (AUC), and the 95% confidence interval (CI) was derived leave-one-out cross-validation. Other diagnosis values (e.g., accuracy, sensitivity, specificity, negative predictive values (NPV), and positive predictive values (PPV)) were measured. The Hosmer-Lemeshow test was used to estimate the goodness-of-fit of models and the calibration plots were performed to test the consistency of models between predicted risk and observed risk in the validation dataset. The decision curve analysis was performed to assess the net benefits based on different threshold probabilities in models. Two-sided *p* values less than 0.05 were considered as a statistical significance. All statistical analysis, model building, and model evaluation were performed in R (version 3.5.2; http://www.r-project.org). Detailed statistical process, R packages, and R functions are described in Supplementary S2.

## Results

### Demographic characteristics

The demographic characteristics of the three datasets are summarized in Table 1. There were no significant differences in terms of clinical record (age, *p* = 0.34; gender, *p* = 0.14; smoking, *p* = 0.16; family history, *p* = 0.49), radiology semantic information (diameter, *p* = 0.54; location, *p* = 0.37; nodules type, *p* = 0.09), and volume values (*p* = 0.60) among three datasets. Additionally, there were no significant differences in final pathological type and surgical type (*p* = 0.08 and *p* = 0.17, respectively) among all datasets.

**Table 1:** Demographic and clinical characteristics of patients on different datasets

Variable	Training (*n* = 331)	Testing (*n* = 143)	Validation (*n* = 149)	*p* value
Age, *n* (%)				
≤ 60	172 (52.0)	74 (51.7)	67 (45.0)	0.34
> 60	159 (48.0)	69 (48.3)	82 (55.0)	
Gender, *n* (%)				
Male	111 (33.5)	45 (31.5)	62 (41.6)	0.14
Female	220 (66.5)	98 (68.5)	87 (58.4)	
Smoking, *n* (%)				
Yes	46 (13.9)	19 (13.3)	30 (20.1)	0.16
No	285 (86.1)	124 (86.7)	119 (79.9)	
Family history, *n* (%)				
Yes	9 (2.7)	6 (4.2)	7 (4.7)	0.49
No	322 (97.3)	137 (95.8)	142 (95.3)	
Final pathology, *n* (%)				
IA	200 (60.4)	86 (60.1)	105 (70.5)	0.08
PM	131 (39.6)	57 (39.9)	44 (29.5)	
Diameter (cm), *n* (%)				
≤ 1	132 (39.9)	54 (37.8)	52 (34.9)	0.54
1.1–2.0	131 (39.6)	51 (35.7)	63 (42.3)	
2.1–3.0	68 (20.5)	38 (26.6)	34 (22.8)	
Location, *n* (%)				
LUL	78 (23.6)	31 (21.7)	26 (17.4)	0.37
LLL	49 (14.8)	28 (19.6)	22 (14.8)	
RUL	127 (38.4)	51 (35.7)	51 (34.2)	
RML	23 (6.9)	10 (7.0)	14 (9.4)	
RLL	54 (16.3)	23 (16.1)	36 (24.2)	
Nodule type, *n* (%)				
pGGN	124 (37.5)	49 (34.3)	37 (24.8)	0.09
PSN	150 (45.3)	71 (49.7)	79 (53.0)	
Solid	57 (17.2)	23 (16.1)	33 (22.1)	
Volume (mm^3^), *n* (%)				
< 500	115 (34.7)	47 (32.9)	43 (28.9)	0.60
500–1000	67 (20.2)	29 (20.3)	27 (18.1)	
> 1000	149 (45.0)	67 (46.9)	79 (53.0)	
Surgical type, *n* (%)				
Lobectomy	256 (77.3)	103 (72.0)	104 (69.8)	0.17
Limited resection	75 (22.7)	40 (28.0)	45 (30.2)	

*IA*, invasive adenocarcinoma; *PM*, preinvasive lesions/minimally invasive adenocarcinomas; *LUL*, left upper lobe; *LLL*, left lower lobe; *RUL*, right upper lobe; *RML*, right middle lobe; *RLL*, right lower lobe; *pGGN*, pure ground-glass nodule; *PSN*, part-solid nodule. *p* values calculated using Pearson’s chi-squared test

Table 2 summarizes cohort differences between patients with final diagnosis of IA and PM without correction for multiple testing. There were significant differences in age, gender, diameter, nodule type, volume, and surgical type (*p* < 0.01 for all), while smoking, family history, and location have no significant differences between two groups. The predictive and diagnosis performance of individual clinical, semantic, and radiomics features are shown in Table 3.

**Table 2:** Demographic and clinical characteristics of patients on IA and PM groups

Variable	IA (*n* = 391)	PM (*n* = 232)	*p* value
Age, *n* (%)			< 0.01*
≤ 60	168 (43.0)	145 (62.5)	
> 60	223 (57.0)	87 (37.5)	
Gender, *n* (%)			< 0.01*
Male	155 (39.6)	63 (27.2)	
Female	236 (60.4)	169 (72.8)	
Smoking, *n* (%)			0.089
Yes	67 (17.1)	28 (12.1)	
No	324 (82.9)	204 (87.9)	
Family history, *n* (%)			0.06
Yes	18 (4.6)	4 (1.7)	
No	373 (95.4)	228 (98.3)	
Diameter (cm), *n* (%)			< 0.01*
≤ 1	57 (14.6)	181 (78.0)	
1.1–2.0	211 (54.0)	34 (14.7)	
2.1–3.0	123 (31.5)	17 (7.3)	
Location, *n* (%)			0.27
LUL	89 (22.8)	46 (19.8)	
LLL	57 (14.6)	42 (18.1)	
RUL	139 (35.5)	90 (38.8)	
RML	27 (6.9)	20 (8.6)	
RLL	79 (20.2)	34 (14.7)	
Nodule type, *n* (%)			< 0.01*
pGGNs	62 (15.9)	148 (63.8)	
PSN	217 (55.5)	83 (35.8)	
Solid	112 (28.6)	1 (0.4)	
Volume (mm^3^), *n* (%)			< 0.01*
< 500	47 (12.0)	158 (68.1)	
500–1000	81 (20.7)	42 (18.1)	
> 1000	263 (67.3)	32 (13.8)	
Surgical type, *n* (%)			< 0.01*
Lobectomy	377 (96.4)	86 (37.1)	
Limited resection	14 (3.6)	146 (62.9)	

*IA*, invasive adenocarcinoma; *PM*, preinvasive lesions/minimally invasive adenocarcinomas; *LUL*, left upper lobe; *LLL*, left lower lobe; *RUL*, right upper lobe; *RML*, right middle lobe; *RLL*, right lower lobe; *pGGN*, pure ground-glass nodule; *PSN*, part-solid nodule. *p < 0.05. *p* values calculated using Pearson’s chi-squared test

**Table 3:** The predictive performance of individual feature on clinical, semantic, and radiomics model on the training dataset

Individual features	ACC (95% CI)	Sensitivity	Specificity	AUC (95% CI)
Clinical				
Age	0.57 (0.51–0.62)	0.28	0.76	0.63 (0.57–0.69)
Gender	0.60 (0.55–0.66)	0	1	0.56 (0.51–0.61)
Smoking	0.60 (0.55–0.66)	0	1	0.55 (0.51–0.58)
Family history	0.60 (0.55–0.66)	0	1	0.50 (0.49–0.52)
Semantic				
Diameter	0.71 (0.66–0.76)	0.60	0.78	0.81 (0.77–0.86)
Location	0.57 (0.52–0.63)	0.02	0.94	0.51 (0.44–0.57)
Nodule type	0.77 (0.72–0.82)	0.69	0.83	0.80 (0.76–0.84)
Radiomics				
LocInt_peakLocal	0.68 (0.64–0.73)	0.60	0.74	0.83 (0.80–0.87)
Wavelet_HLL_Stats_max	0.68 (0.63–0.72)	0.59	0.73	0.85 (0.82–0.89)
GLRLM_LGRE	0.76 (0.72–0.80)	0.68	0.81	0.90 (0.87–0.92)
Wavelet_LLL_Stats_cov	0.73 (0.68–0.77)	0.63	0.79	0.87 (0.84–0.90)

*ACC*, accuracy; *AUC*, area under curve; *CI*, confidence interval

### Feature selection

A total of 1322 radiomics feature were extracted. After analysis of features stability to ROI variations, 325 features with ICC values less than 0.8 were removed (Supplementary S3). Ten features were removed due to little variance and 875 features (including volume) were removed due to high correlation with other features, leaving 112 features. The frequency of the top ten selected features and the distribution of the AUC values of the generated 10-feature models for the 1000 iterations are shown in Supplementary S4. When applying the pipeline to data with randomized outcomes, the mean AUC upon 1000 iterations for 5 feature models was 0.53 (Supplementary S4).

**Fig. 3:**
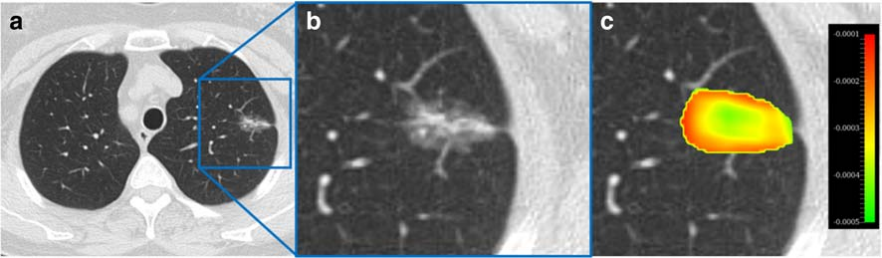
**a** CT axial view of a pulmonary nodule; **b** zoomed in view; **c** feature map overlaid on the zoomed in CT

Of the features selected with high frequency, the top 5 features (with frequencies 850, 817, 805, 727, and 343 per 1000 iterations) were pre-selected using our methodology. One of these features (Wavelet_LHH_GLDZM_LIE) was removed due to a high correlation with tumor volume (*r* = − 0.84), arriving at a model with four robust radiomics features. The feature map is shown in Fig. 3.

### AUC of radiomics vs. clinical, semantic, volume, and FS

The radiomics model performed well when classifying between IA and PM with an AUC of 0.89 (95% CI, 0.86–0.93), 0.89 (95% CI, 0.83–0.94), and 0.88 (95% CI, 0.81–0.94) on the training, testing, and validation datasets, respectively. There was no significant difference between the AUC values of the radiomics and FS models on the three datasets (training: AUC = 0.90 (95% CI, 0.87–0.93), *p* = 0.82; testing: AUC = 0.93 (95% CI, 0.88–0.97), *p* = 0.20; validation: AUC = 0.92 (95% CI, 0.87–0.96), *p* = 0.29). The radiomics models performed better than the clinical model on all datasets, and outperformed semantic and volume models only in the training dataset, while no significant differences were seen on the testing and validation dataset (Table 4; Supplementary S5).

**Table 4:** The detailed AUC values and *p* values among models on the three datasets

Dataset	Model	AUC	95% CI	P1	P2	P3
Training	Clinical	0.58	0.52–0.65	< 0.01*		
	Semantic	0.85	0.81–0.89	0.01*		
	Volume	0.84	0.80–0.88	0.01*		
	FS	0.90	0.87–0.93	0.82	0.58	< 0.01*
	Radiomics	0.89	0.86–0.93			
	RV	0.90	0.87–0.94		0.18	< 0.01*
	CSRV	0.91	0.88–0.94			
	FSV	0.94	0.91–0.96			0.06
	FSRV	0.96	0.94–0.98			
	CSFSRV	0.96	0.94–0.98		< 0.01*	0.74
Testing	Clinical	0.55	0.45–0.65	< 0.01*		
	Semantic	0.85	0.78–0.92	0.28		
	Volume	0.87	0.81–0.93	0.54		
	FS	0.93	0.88–0.97	0.20	0.21	0.01*
	Radiomics	0.89	0.83–0.94			
	RV	0.88	0.82–0.93		0.10	< 0.01*
	CSRV	0.89	0.84–0.94		0.21	
	FSV	0.98	0.96–1			0.50
	FSRV	0.97	0.94–1			
	CSFSRV	0.97	0.94–0.99		< 0.01*	0.25
Validation	Clinical	0.61	0.51–0.72	< 0.01*		
	Semantic	0.87	0.81–0.92	0.75		
	Volume	0.93	0.88–0.98	0.16		
	FS	0.92	0.87–0.96	0.29	0.97	0.01*
	Radiomics	0.88	0.81–0.94			
	RV	0.91	0.86–0.96		0.71	0.03*
	CSRV	0.92	0.87–0.96			
	FSV	0.97	0.94–0.99			0.62
	FSRV	0.96	0.93–0.99			
	CSFSRV	0.96	0.94–0.99		0.01*	0.30

*FS*, frozen section; *RV*, radiomics combining with volume; *CSRV*, radiomics combing with clinical, semantic, and volume; *FSV*, frozen section combining with volume; *FSRV*, frozen section combining with radiomics and volume; *CSFSRV*, radiomics combining with clinical, semantic, volume, and frozen section; *AUC*, the area under the curve; *CI*, confidence interval. *p < 0.05; P1 = *p* values between radiomics and other models; P2 = *p* values between CSRV and other models; P3 = *p* values between FSRV and other models. *p* values calculated using roc test by Delong method

### AUC of CSRV vs. FS, RV, and CSFSRV

Compared with the FS and RV model, the CSRV model did not show improved AUC value (training: AUC = 0.91, 95% CI 0.88–0.94; testing: AUC = 0.89, 95% CI 0.84–0.94; validation AUC = 0.92, 95% CI 0.87–0.96; *p* > 0.05), while it was significantly worse than the CSFSRV model (training: AUC = 0.96, 95% CI 0.94–0.98, *p* < 0.01; testing AUC = 0.97, 95% CI 0.94–0.99, *p* < 0.01; validation: AUC = 0.96, 95% CI 0.94–0.99, *p* = 0.01).

### AUC of FSRV vs. RV, FS, FSRV, and CSFSRV

Adding FS into the radiomics and volume (FSRV) model improved the classification performance between IA and PM (training: AUC = 0.96, 95% CI 0.94–0.98; testing: AUC = 0.97, 95% CI 0.94–1; validation: AUC = 0.96, 95% CI 0.93–0.99), which significantly outperforms both RV and FS alone on all three datasets. There were no significantly differences between FSRV and both FSV and CSFSRV models on all datasets (Table 4; Supplementary S6).

### Accuracy of models

The FS model was able to discriminate between IA and PM on the training, testing, and validation dataset with an accuracy of 0.90, 0.92, and 0.90, respectively. The radiomics model had a higher accuracy than other single-factor models including clinical, semantic, and volume on all three datasets (0.82, 0.79, and 0.85). When FS combined with RV (0.83, 0.80, and 0.87), the resulting FSRV model showed an improved accuracy with values of 0.91, 0.94, and 0.93 on the three datasets, outperforming FSV (0.89, 0.92, and 0.91). When the FS model is enriched with CSRV (0.83, 0.83, and 0.87), the resulting CSFSRV had lower accuracy than the FSRV model in the testing and validation (0.92 and 0.91) and same accuracy in the training (0.91). The detailed accuracy, sensitivity, specificity, PPV, and NPV are summarized in Table 5.

**Table 5:** The detailed diagnosis values of models on three datasets

Dataset	Model	Accuracy (95% CI)	Sensitivity	Specificity	PPV	NPV
Training	Clinical	0.63 (0.58–0.68)	0.31	0.84	0.56	0.65
	Semantic	0.79 (0.74–0.83)	0.79	0.79	0.71	0.85
	Volume	0.66 (0.61–0.71)	0.61	0.69	0.56	0.73
	FS	0.90 (0.86–0.93)	0.91	0.89	0.84	0.94
	Radiomics	0.82 (0.78–0.86)	0.79	0.85	0.77	0.86
	RV	0.83 (0.79–0.87)	0.79	0.86	0.78	0.86
	CSRV	0.83 (0.78–0.87)	0.80	0.85	0.77	0.87
	FSV	0.89 (0.85–0.92)	0.89	0.89	0.84	0.93
	FSRV	0.91 (0.88–0.94)	0.90	0.92	0.88	0.93
	CSFSRV	0.91 (0.87–0.94)	0.89	0.92	0.88	0.93
Testing	Clinical	0.63 (0.54–0.71)	0.23	0.90	0.59	0.64
	Semantic	0.78 (0.71–0.85)	0.68	0.85	0.75	0.80
	Volume	0.69 (0.60–0.76)	0.61	0.73	0.60	0.74
	FS	0.92 (0.87–0.96)	0.95	0.91	0.87	0.96
	Radiomics	0.79 (0.71–0.85)	0.70	0.85	0.75	0.81
	RV	0.80 (0.73–0.87)	0.74	0.85	0.76	0.83
	CSRV	0.83 (0.75–0.88)	0.77	0.86	0.79	0.85
	FSV	0.92 (0.87–0.96)	0.95	0.91	0.87	0.96
	FSRV	0.94 (0.88–0.97)	0.86	0.99	0.98	0.91
	CSFSRV	0.92 (0.87–0.96)	0.84	0.98	0.96	0.90
Validation	Clinical	0.70 (0.62–0.77)	0.18	0.91	0.47	0.73
	Semantic	0.79 (0.72–0.85)	0.68	0.84	0.64	0.86
	Volume	0.79 (0.72–0.85)	0.66	0.85	0.64	0.86
	FS	0.90 (0.84–0.94)	0.95	0.88	0.76	0.98
	Radiomics	0.85 (0.78–0.90)	0.61	0.94	0.82	0.85
	RV	0.87 (0.81–0.92)	0.68	0.95	0.86	0.88
	CSRV	0.87 (0.81–0.92)	0.73	0.93	0.82	0.89
	FSV	0.91 (0.85–0.95)	0.95	0.89	0.78	0.98
	FSRV	0.93 (0.87–0.96)	0.93	0.92	0.84	0.97
	CSFSRV	0.91 (0.86–0.95)	0.89	0.92	0.83	0.95

*FS*, frozen section; *RV*, radiomics combining with volume; *CSRV*, radiomics combining with clinical, semantic, and volume; *FSV*, frozen section combining with volume; *FSRV*, frozen section combining with radiomics and volume; *CSFSRV*, radiomics combining with clinical, semantic, volume, and frozen section; *PPV*, positive predictive values; *NPV*, negative predictive values; *CI*, confidence interval

### Calibration, decision curve analysis, RQS, and TRIPOD

**Fig. 4:**
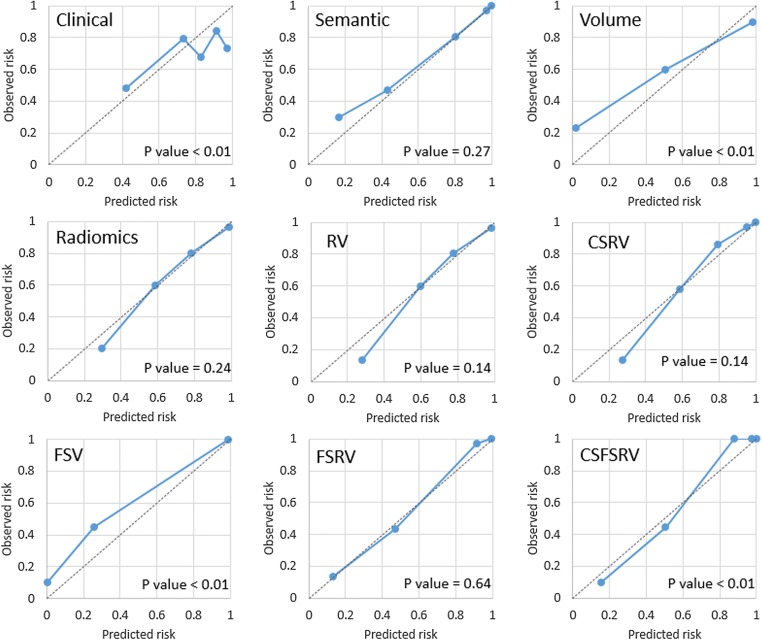
The calibration plots of the single and complex models on the validation dataset

**Fig. 5:**
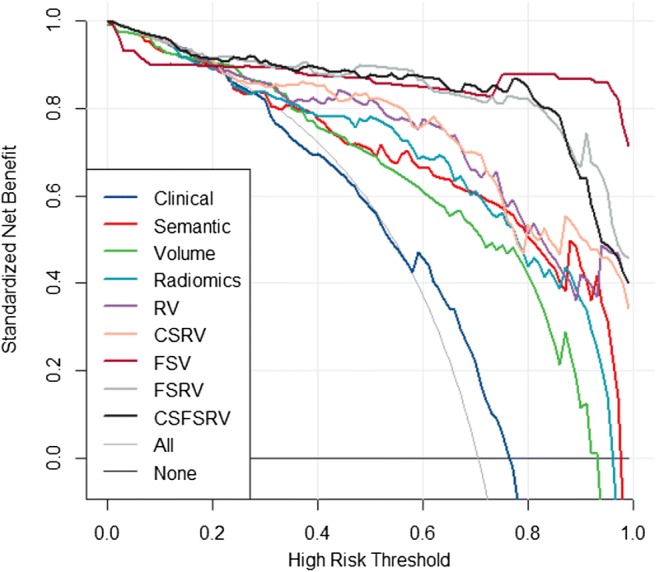
The decision curve of models performed on the validation dataset

Among all models, the semantic, radiomics, RV, and FSRV models showed good calibrations with *p* values of 0.27, 0.24, 0.14, and 0.64, respectively, in the validation dataset (calibration plots depicted in Fig. 4). From the decision curve analysis on the validation dataset, we found that the clinical and volume models alone perform worse than semantic, radiomics, RV, and CSRV models. Models combining FS with other modalities (i.e., FSV, FSRV, and CSFSRV) perform the best. However, it seems that there is no obvious difference between the FSV, FSRV, and CSFSRV models (Fig. 5). The RQS points and total score of this study were 17 and 47.2%, respectively. We concluded this signature could be classified as TRIPOD 3 (Supplementary S6).

## Discussion

In this multicenter study, multiple univariate and multivariate binary classification models have been built to distinguish IA from PM using combinations of radiomics features as well as clinical features, semantic features, volume, and frozen section results. A method to select quantitative imaging features that are robust to spurious distributions of patient subgroups within the cohort as well as being mostly independent of the ROI volume is presented. The performance of the radiomics classifier was compared with models informed by clinical or semantic features, volume, and frozen section. The performance of the multifactorial FSRV diagnostic model was also compared with FS, RV, FSV, and CSFSRV models. Our results show that a multifactorial model based on radiomics features combined with FS and volume had excellent classification performance and diagnostic accuracy, suggesting that it can potentially be employed to gauge the risk of invasiveness in malignant pulmonary nodules.

Smoking is one of the most important risk factors for developing lung cancer, which is strongly correlated with the number of years and the amount of tobacco smoked [26]. Moreover, a population-based prospective study indicated that the risk factor for developing lung cancer increases with age and with a family history of lung cancer for female patients [27]. However, in this study, only age and gender significantly differ between cohorts diagnosed with IA and PM, with males older than 60 years having a significantly higher probability to be diagnosed with IA. Age has been reported elsewhere to increase the risk factor of IA diagnosis, while gender differences in the adenocarcinoma spectrum need further study [8–10]. Our results also show that a model informed purely on clinical variables has low sensitivity and relatively high specificity for the identification of IA, which may lead to moderate accuracy for diagnosis and low benefit from decision curve. This result, however, should be interpreted with caution, because clinical variables are varied in different populations.

Another study also looked at semantic features, proposing that pulmonary nodules with a larger diameter, located in the upper lobe, spiculation, and PSN (part-solid nodule) had a higher probability to be malignant [27]. However, it has been shown that semi-automated volume analysis is a more robust method than a simple measurement of the diameter to measure the size of the pulmonary nodule [28], and spiculation is an uncommon feature in early-stage lung cancer [8]. Our study finds that nodule diameter and nodule type are significantly different between cohorts diagnosed with IA and PM, with nodules with smaller diameter and pure GGN types increasing the probability of PM diagnosis. These two semantic features by themselves, as well as the semantic model, show high AUC and accuracy values for prediction and diagnosis of IA. Overall, our results indicate both a semantic feature model and a lesion volume model show similar predictive performance compared with radiomics, while radiomics has higher accuracy than semantic and volume models.

It is important also to point out that the ground truth used for diagnosis in this study is fairly unique as resections are not generally considered for pGGNs in guidelines in most countries outside of Asia where pGGNs are followed up until a solid component appears or the tumor progresses [29]. Moreover, pGGN adenocarcinomas are more common in low-risk Asian females than other populations, and the patients more often request surgery. Around 34% of nodules in this study are pGGNs, 30% of which are confirmed as IA, which may reflect doctors’ and patients’ more positive attitudes towards surgery.

In our study, the CT-based radiomics model shows a similar predictive performance with FS in distinguishing IA from PM. Selected features (Wavelet_HLL_Stats_max, Wavelet_LLL_Stats_cov, and LocInt_peakLocal) reflect the distribution of intensity values within the ROI, and another selected feature (GLRLM_LGRE) describes the heterogeneity of the density within the ROI [23]. Lim et al found that the mean density differs between IA and non- or minimally IA [8]. Moreover, a previous study reported that IA tends to appear more heterogeneous on CT images than PM [30]. Therefore, we hypothesize that radiomics features describing density and heterogeneity are related to tumor biology and pathology and are an excellent predictor for identification of IA [25].

CT and positron emission tomography radiomics studies have shown predictive features could be a surrogate of lesion volume and knowledge of which features correlate highly with volume is therefore important [31–33]. Upon volume correlation analysis, we excluded one feature that correlated highly with volume and found no change in model performance. The volume was embedded into the radiomics signature since radiomics is synonymous with quantitative imaging; features that contribute to model performance should not be excluded a priori. In this study, a radiomics plus volume model (RV) showed slight improvement of accuracy compared with the radiomics-alone model, and it had similar AUC and accuracy values as the CSRV model. In addition, we found that our models employing radiomics (i.e., radiomics alone, RV, and CSRV) had similar predictive performance (AUC) as the frozen section models. However, the accuracy of these models was lower than that of FS.

Although the FS can be a precise diagnostic method to guide intraoperative resection procedures for lung adenocarcinoma, it remains difficult to recommend a definitive assessment by FS alone [34]. Borczuk suggested that combining clinical and radiologic information with FS could reduce diagnostic errors [35]. Our results show no significant difference in the AUC values between the FSRV and FSV models, but the former model has better accuracy and calibration. Furthermore, we found that the AUC of the CSFSRV model is not significantly different from that of the FSRV model, did not increase the accuracy, and got bad calibration. In addition, the decision curve indicates that the models containing FS all had better performance than the models without FS. Therefore, we conclude that the addition of radiomics (with volume) to FS analysis potentially creates a substantial biomarker for assessing the risk of invasive adenocarcinomas and could be applied in clinical practice.

Nevertheless, this study has certain limitations. First, because of the retrospective data collection, selection bias is unavoidable. Further prospectively international investigation as a registered clinical trial is paramount. Second, different population cohorts, tumor morphology, and CT parameters are known to influence the results of radiomics features [36]. Further external validation datasets are desired to verify the reliability of our model, especially including diverse cohorts to fully capture phenotype heterogeneity. Third, the ROIs were contoured manually, which is time-consuming and highly prone to error. Therefore, a reliable and robust automatic segmentation tool is necessary to address this issue [37], also taking into account, e.g., peritumoral and normal tissue, to increase the accuracy of quantitative image-based models. Fourth, the accuracy and specificity of the FS analysis in our cohort were lower than the results from previous studies [6, 11]. We speculate that we included more small size and GGN cases, which have lower accuracy than larger tumors as most studies found [6, 11, 12]. Future prospects include prospective validation and deep learning methods for automatic segmentation and in combination with the ones described in this study, novel parametric imaging techniques. While this work focuses on the correlation of radiomics features with the underlying biology (histology), future work will also focus on the prediction of clinical outcomes directly, such as overall survival, progression free survival, or response to therapy.

In conclusion, a radiomics signature can be employed as a preoperative tool to distinguish invasive adenocarcinoma from preinvasive lesions or MIA. Furthermore, a multifactorial model combining radiomics with FS analysis is a potential biomarker for assessing the risk of invasive adenocarcinoma during surgery, and this model could help the therapeutic strategy for patients with pulmonary nodules.

## Electronic supplementary material

ESM 1 (DOCX 454 kb)

## Abbreviations

AAH: Atypical adenomatous hyperplasia
AIS: Adenocarcinoma in situ
AUC: Area under the curve
CI: Confidence interval
CT: Computed tomography
FS: Frozen section
GGN: Ground-glass nodule
IA: Invasive adenocarcinoma
ICC: Intra-/inter-class correlation coefficients
MIA: Minimally invasive adenocarcinoma
NPV: Negative predictive values
pGGN: Pure ground-glass nodule
PM: Preinvasive lesions or minimally invasive adenocarcinomas
PPV: Positive predictive values
PSN: Part-solid nodule
ROC: Receiver operating characteristic
ROI: Regions of interest
ROV: Radiomics plus volume model
TRIPOD: Transparent Reporting of a Multivariable Prediction Model for Individual Prognosis or Diagnosis
