# Supplementary material for: Preoperative CT-based radiomics combined with intraoperative frozen section is predictive of invasive adenocarcinoma in pulmonary nodules: a multicenter study
Source: Eur Radiol. 2020 Jan 31;30(5):2680–91. doi: 10.1007/s00330-019-06597-8 (PMC7160197; doi:10.1007/s00330-019-06597-8)
Supplement: Supplementary file 1 — (DOCX 454 kb) [file 330_2019_6597_MOESM1_ESM.docx]

**Supplementary S1: CT acquisition parameters of hospitals**

| Parameters | Hospital1 | Hospital2 | Hospital3 |
| --- | --- | --- | --- |
| Manufacture | Siemens | Siemens | Siemens |
| Acquisition type | Helical | Helical | Helical |
| Tube voltage | 100 kVp | 120 kVp | 120 kVp |
| Tube current | automatically | automatically | automatically |
| Pitch | 1.2-1.5 | 0.75-1 | 1.4 |
| Collimation | 0.6 | 0.6 | 0.6 |
| Matrix | 512 × 512 | 512 × 512 | 512 × 512 |
| Pixel spacing | 0.6-1.0 mm | 0.64-0.96 mm | 0.75 mm |
| Reconstruction kernel | B50f; B60f | B60f | B50f |
| Thickness | 1 mm | 1 mm | 1.5 mm |

Note: Hospital 1 (Affiliated Zhongshan Hospital of Dalian University), Hospital 2 (The Second Affiliated Hospital of Dalian Medical University, and Hospital 3 (The Fifth Hospital of Dalian).

**Supplementary S2: Statistical process, R packages, and R functions.**

Pearson's Chi-Squared test was used by “chisq.test” for the statistical analysis of essential demographic characteristics. Reliability and reproducibility of segmentations were estimated by calculating the intra-/inter-class correlation (ICC) values in the package “irr”. The correlation within features was performed by using Spearman's rank correlation coefficient in the package “cor”. The process of the spilt sample, recursive feature elimination (using function treebag), and cross-validation are based on the package “caret”. The modeling process is done by “randomForest”. The AUC with 95%CI was measured by using the package “pROC”. The package “roc.test” was used to test the statistical significance among different models. The package “confusionMatrix” was used to calculate other prediction measures, including accuracy, sensitivity, specificity, NPV, and PPV. The package “roc.test” was used to test the statistical significance among different models. The function “plotCalibration” was used to plot the calibration and perform the Hosmer-Lemeshow test. The package “rmda” was used to draw the decision curve.

**Supplementary S3: Histograms of ICC used to assess the robustness of features from 50 cases segmented by two radiologists.**

A, Histogram of the intra-class correlation coefficients. B, Histogram of the inter-class correlation coefficients. The features with ICCs value less than 0.8 were removed.


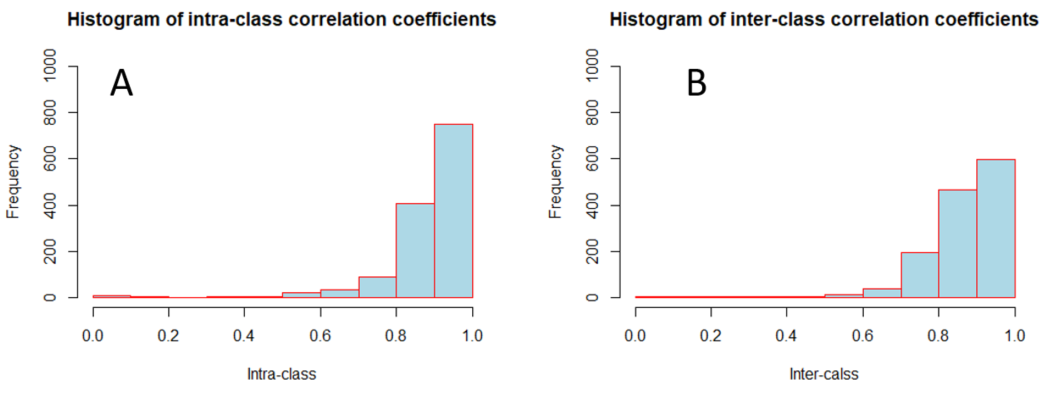


**Supplementary S4: *The results of features frequency and AUC values.***

A, The frequency of the top ten selected features for each radiomics model upon 1000 iterations. B, The kernel density of AUC values for each radiomics model upon 1000 iterations. C, The frequency histogram of AUC values for each radiomics model with randomized outcomes upon 1000 iterations.


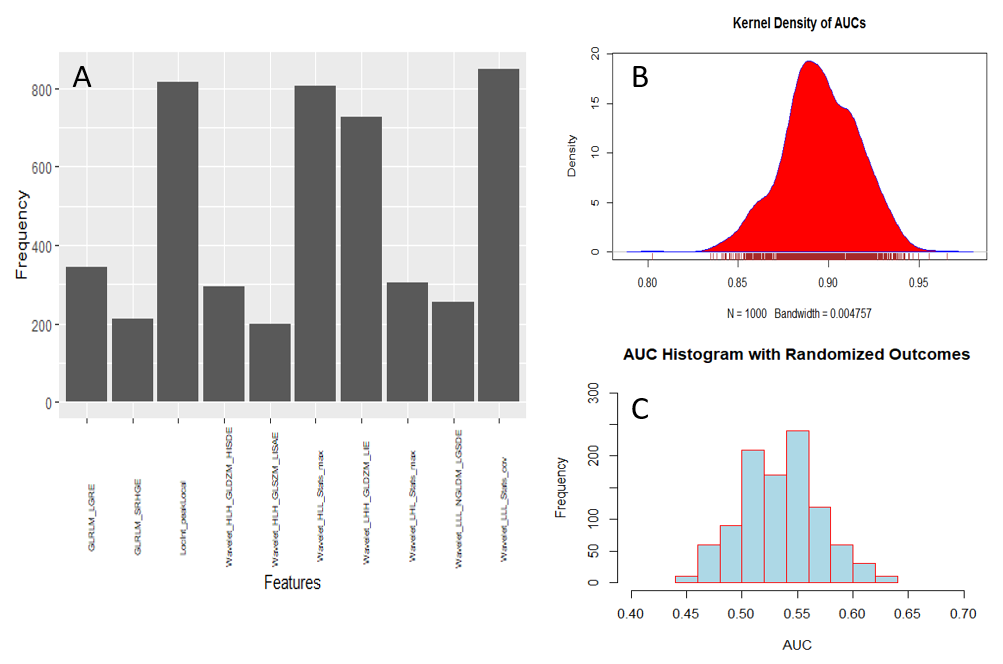


**Supplementary S5*: ROC curves of Models***

The upper three panels showed the ROCs and AUC values (with 95% confidence interval) of radiomics, clinical, semantic, volume, and frozen section models on the training, testing and validation datasets; and the lower three graphs exhibited the ROCs and AUC values of RV, CSRV, frozen section, FSRV, FSV, and CSFSRV models.


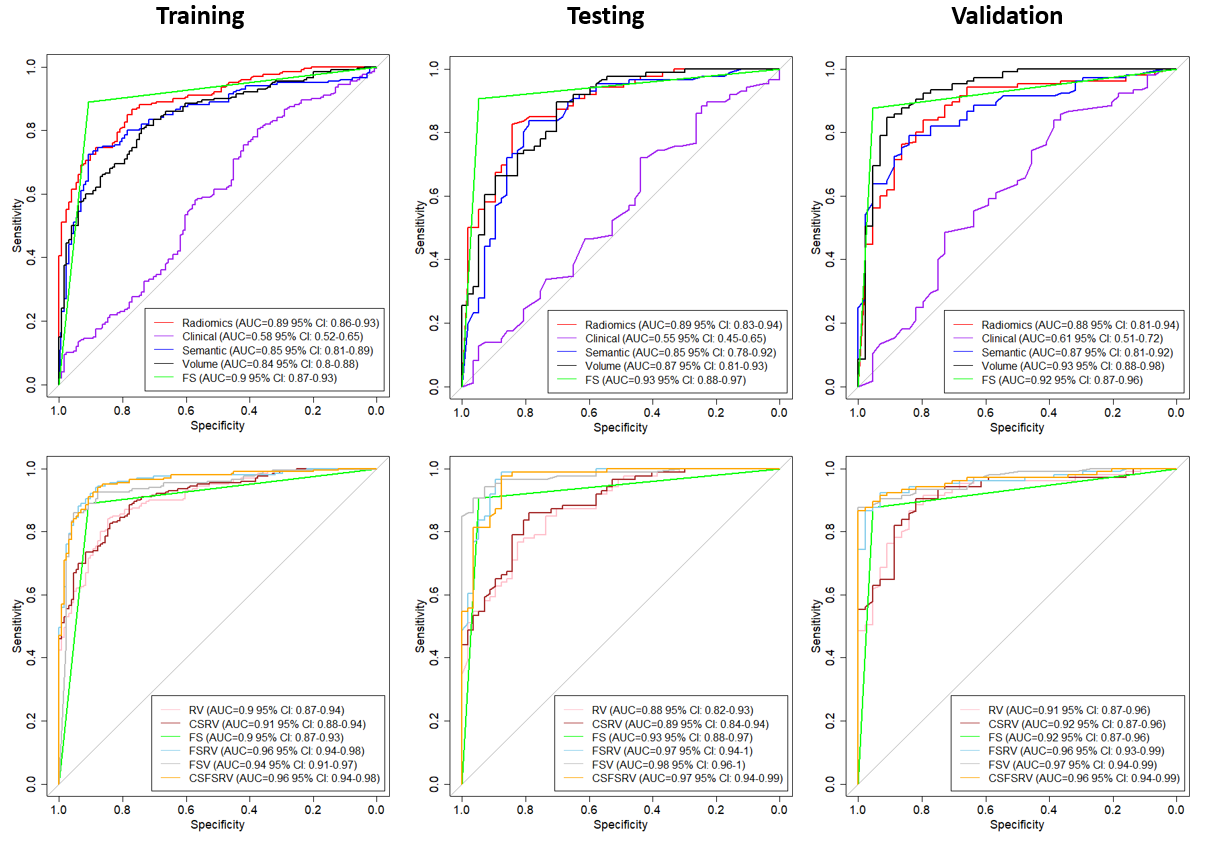


**Supplementary S6: TRIPOD checklist**

| **Section/Topic** | **Item** |  | **Checklist Item** | **Page** |
| --- | --- | --- | --- | --- |
| **Title and abstract** | | | | |
| Title | 1 | D;V | Identify the study as developing and/or validating a multivariable prediction model, the target population, and the outcome to be predicted. | 1 |
| Abstract | 2 | D;V | Provide a summary of objectives, study design, setting, participants, sample size, predictors, outcome, statistical analysis, results, and conclusions. | 1 |
| **Introduction** | | | | |
| Background and objectives | 3a | D;V | Explain the medical context (including whether diagnostic or prognostic) and rationale for developing or validating the multivariable prediction model, including references to existing models. | 3 |
|  | 3b | D;V | Specify the objectives, including whether the study describes the development or validation of the model or both. | 3 |
| **Methods** | | | | |
| Source of data | 4a | D;V | Describe the study design or source of data (e.g., randomized trial, cohort, or registry data), separately for the development and validation data sets, if applicable. | 4 |
|  | 4b | D;V | Specify the key study dates, including start of accrual; end of accrual; and, if applicable, end of follow-up. | 4 |
| Participants | 5a | D;V | Specify key elements of the study setting (e.g., primary care, secondary care, general population) including number and location of centres. | 4 / Table1 |
|  | 5b | D;V | Describe eligibility criteria for participants. | 4 |
|  | 5c | D;V | Give details of treatments received, if relevant. | - |
| Outcome | 6a | D;V | Clearly define the outcome that is predicted by the prediction model, including how and when assessed. | 5 |
|  | 6b | D;V | Report any actions to blind assessment of the outcome to be predicted. | 5 |
| Predictors | 7a | D;V | Clearly define all predictors used in developing the multivariable prediction model, including how and when they were measured. | 5 |
|  | 7b | D;V | Report any actions to blind assessment of predictors for the outcome and other predictors. | 5 |
| Sample size | 8 | D;V | Explain how the study size was arrived at. | 4/ Fig 1 |
| Missing data | 9 | D;V | Describe how missing data were handled (e.g., complete-case analysis, single imputation, multiple imputation) with details of any imputation method. | - |
| Statistical analysis methods | 10a | D | Describe how predictors were handled in the analyses. | 5-6 |
|  | 10b | D | Specify type of model, all model-building procedures (including any predictor selection), and method for internal validation. | 6 |
|  | 10c | V | For validation, describe how the predictions were calculated. | 6 |
|  | 10d | D;V | Specify all measures used to assess model performance and, if relevant, to compare multiple models. | 7/S2 |
|  | 10e | V | Describe any model updating (e.g., recalibration) arising from the validation, if done. | - |
| Risk groups | 11 | D;V | Provide details on how risk groups were created, if done. | - |
| Development vs. validation | 12 | V | For validation, identify any differences from the development data in setting, eligibility criteria, outcome, and predictors. | 7/ Table 1 |
| **Results** | | | | |
| Participants | 13a | D;V | Describe the flow of participants through the study, including the number of participants with and without the outcome and, if applicable, a summary of the follow-up time. A diagram may be helpful. | Fig 1 |
|  | 13b | D;V | Describe the characteristics of the participants (basic demographics, clinical features, available predictors), including the number of participants with missing data for predictors and outcome. | 7/Table 1 |
|  | 13c | V | For validation, show a comparison with the development data of the distribution of important variables (demographics, predictors and outcome). | 7, 8/Table 1 |
| Model development | 14a | D | Specify the number of participants and outcome events in each analysis. | Table 3 |
|  | 14b | D | If done, report the unadjusted association between each candidate predictor and outcome. | Table 2 |
| Model specification | 15a | D | Present the full prediction model to allow predictions for individuals (i.e., all regression coefficients, and model intercept or baseline survival at a given time point). | Table 3 |
|  | 15b | D | Explain how to use the prediction model. | 8 |
| Model performance | 16 | D;V | Report performance measures (with CIs) for the prediction model. | 8,9/Table 2,3 |
| Model-updating | 17 | V | If done, report the results from any model updating (i.e., model specification, model performance). | - |
| **Discussion** | | | | |
| Limitations | 18 | D;V | Discuss any limitations of the study (such as nonrepresentative sample, few events per predictor, missing data). | 11 |
| Interpretation | 19a | V | For validation, discuss the results with reference to performance in the development data, and any other validation data. | 10 |
|  | 19b | D;V | Give an overall interpretation of the results, considering objectives, limitations, results from similar studies, and other relevant evidence. | 11 |
| Implications | 20 | D;V | Discuss the potential clinical use of the model and implications for future research. | 11 |
| **Other information** | | | | |
| Supplementary information | 21 | D;V | Provide information about the availability of supplementary resources, such as study protocol, Web calculator, and data sets. | S1-S5 |
| Funding | 22 | D;V | Give the source of funding and the role of the funders for the present study. | Title page |
